# Carbapenemase-Producing Enterobacterales in Pets: A New Threat From Veterinary Clinics

**DOI:** 10.1093/cid/ciaf544

**Published:** 2025-09-26

**Authors:** Vincent Perreten, Andrea Endimiani

**Affiliations:** Institute of Veterinary Bacteriology, University of Bern, Bern, Switzerland; Institute for Infectious Diseases, University of Bern, Bern, Switzerland

**Keywords:** animals, carbapenemases, transmission, humans, veterinary

**(See the Major Article by DeStefano et al on pages e1138–44.)**

The spread of carbapenemase-producing Enterobacterales (CPE) represents a global public health concern due to its significant clinical implications [1, 2]. Individuals infected and/or colonized with CPE can transmit these pathogens to others, which may contribute to their expansion in nonhuman settings, including animals, the environment, and the food chain [3–6].

CPE has been increasingly observed in animals, including companion animals hospitalized at specialized veterinary clinics [7]. CPE recovered from such institutions harbor the same carbapenemase genes (eg, *bla*_OXA-48/OXA-181_, *bla*_NDM-5_, *bla*_KPC-2_) and may belong to the same sequence types (STs; eg, *Klebsiella pneumoniae* ST11, *Escherichia coli* ST410/ST167) that circulate in humans [7–12]. Of particular concern, clonal spread of CP *E. coli* within veterinary hospitals can lead to large and prolonged outbreaks that affect pets and the hospital environment [13–16]; transmission to hospital personnel may also occur [16–18]. However, despite these observations, data on the occurrence and risk of CPE transmission from hospitalized pets to their owners remain scarce and need to be obtained in order to apply appropriate measures to mitigate the global spread of these pathogens.

In this issue of *Clinical Infectious Diseases*, DeStefano et al describe 3 cases of human infection or colonization with New Delhi metallo-β-lactamase (NDM)-5–producing *E. coli* (NDM-5-*Ec*) that were highly related to those isolated during an outbreak at a Massachusetts veterinary hospital. The 3 patients had either recurrent urinary tract infections or lymphoma but shared no recognizable epidemiological links (eg, hospitalization in the same institution), apart from residing in the same county and owning dogs or cats taken to the same veterinary facility within 2 months prior to their infection. Illumina-based whole-genome sequencing (WGS) analysis revealed that all NDM-5-*Ec* isolated from this veterinary hospital, including from hospitalized pets (n = 9) and the environment (n = 17), as well as from the 3 patients, were highly related to each other (≤10 single nucleotide polymorphisms [SNPs]) [19], belonged to ST162, and harbored the *bla*_NDM-5_ on an IncX3 plasmid.

NDM-5-*Ec* strains have been increasingly reported in humans worldwide, including as the cause of hospital outbreaks. Notably, these pathogens frequently belong to the high-risk ST167, ST361, ST405, ST410, ST617, and ST648 [20–24]. Regarding the veterinary setting, sporadic NDM-5-*Ec* isolates have been reported globally [7]. However, only 1 large hospital outbreak caused by an ST167 NDM-5-*Ec* has been described (in Pennsylvania) [15], while smaller epidemic events have been reported in China (ST405, ST410, ST453) and Switzerland (ST167) [16, 18, 25, 26].

In this scenario, the report by DeStefano et al of a veterinary hospital outbreak in Massachusetts due to NDM-5-*Ec* is not surprising, although the bacterial host belonged to the rare ST162. Unfortunately, the authors analyzed only a partial sequence (9082-bp) of the *bla*_NDM-5_-IncX3 plasmid carried by the clustered NDM-5-*Ec*. In fact, *bla*_NDM-5_ can be found on IncX3 plasmids with sizes that vary from 30 kb to 70 kb [27]. The lack of a comparative plasmid sequence analysis limits the ability to assess potential epidemiological links with plasmids that spread locally or in other countries in both human and nonhuman settings [28]. In our view, comprehensive plasmid-level information (including conjugation frequency) is crucial, as carbapenemase dissemination can occur not only via clonal expansion of the bacterial host but also through horizontal transfer of epidemic plasmids to different lineages (STs) or Enterobacterales species [9–11].

As anticipated, the ST162 NDM-5-*Ec* isolated from the veterinary clinic setting and the 3 humans were nearly identical. However, authors stated that WGS analysis did not identify additional closely related (ie, ≤50 SNPs) NDM-5-*Ec* strains among human clinical samples in Massachusetts.

In Switzerland, we investigated the potential link between an ST410 OXA-181–producing *E. coli* (OXA-181-*Ec*) that caused a veterinary clinic outbreak and *E. coli* isolates of the same ST recovered from humans during the same period. Most strains carried the same 51-kb IncX3 *bla*_OXA-181_-positive plasmid but differed in SNP-based genetic distance (≥131 SNPs) and antimicrobial resistance gene content, indicating independent evolution [11]. We also compared OXA-48–producing *E. coli* (OXA-48-*Ec*) strains isolated from hospitalized pets, the hospital environment, humans, and rivers. These strains exhibited high genetic diversity, with only a few sharing the same ST. However, the epidemic 63-kb *bla*_OXA-48_-IncL plasmid was frequently detected across lineages, indicating the trans-sectoral dissemination of such a mobile genetic element [29]. Of note, analysis of soil samples from a public park near the veterinary clinic where OXA-181-*Ec* and OXA-48-*Ec* had been detected did not reveal any CP *E. coli* strains [30]. Moreover, in a nationwide river survey that assessed the occurrence of CPE, the epidemic 51-kb *bla*_OXA-181_-harboring IncX3 plasmid was identified in both *E. coli* ST410 and non-ST410 [6]. Finally, in a prospective, longitudinal, observational study that involved 50 dog and cat owners (n = 271) who presented to 5 veterinary institutions, CPE owner–pet co-carriage was never observed [31]. Taken together, these surveys from Switzerland and Massachusetts suggest that clonal spillover of CP *E. coli* from veterinary hospital settings to humans and the environment appears to be limited, whereas the dissemination of epidemic plasmids deserves further attention. Whether these conclusions are universally applicable should be addressed through large international studies that simultaneously compare CPE isolates from humans, veterinary settings, and environmental sources.

Remarkably, although the dogs and cat that belonged to the 3 human cases were not tested for CPE, DeStefano et al hypothesized that these animals became colonized during hospitalization and subsequently transmitted CPE to their owners after discharge. While alternative explanations were considered and thoroughly discussed by the authors, we agree that this remains the most plausible scenario, especially in light of the sampling timeline and additional information obtained regarding the patients and their pets. However, the study lacks formal proof that the pets served as transmission vehicles for the ST167 NDM-5-*Ec* to humans. Such evidence can only be obtained by detecting the CPE in both owners and their pets, followed by precise WGS strain analysis at both the chromosomal and plasmid levels. To our knowledge, only the 3 studies discussed below have applied this approach.

Grönthal et al (Finland) reported identical ST167 NDM-5-*Ec* strains shared between 2 dogs with otitis and their owner in the household [4]. Our group (Switzerland) described 2 healthy employees who were colonized with ST410 OXA-181-*Ec* and ST167 NDM-5-*Ec* strains undistinguishable from those that caused epidemic events at 2 veterinary clinics [17]. Wang et al (China) showed that an identical (0 SNPs) ST453 NDM-5-*Ec* colonized the intestinal tract of 1 hospitalized dog and 2 of its healthcare providers [18]. Nevertheless, in these 3 studies, the trajectory of transmission from animals to humans was not formally demonstrated, as human negative samples prior to animal CPE detection were not available.

In our opinion, CPE transmission from previously hospitalized dogs or cats to their owners is likely to occur; however, evidence remains very limited, warranting further investigation. In this context, it should be noted that pets discharged from clinics may remain colonized with CP *E. coli* for extended periods, in some cases, up to 3 months [13]. On the other hand, veterinary clinics clearly constitute important reservoirs for the selection and dissemination of high-risk CPE strains and their associated epidemic plasmids [7–16]. Moreover, veterinary facilities that face high prevalence of CPE may contribute to the spread of these difficult-to-treat pathogens in the community through colonization of staff [17, 18]. Therefore, as discussed by DeStefano et al, veterinary institutions should implement countermeasures to avoid the uncontrolled dissemination of CPE and the risk of seeing endemic strains emerge within the companion animal population.

In view of a One Health approach, all stakeholders (eg, human/veterinary clinicians, epidemiologists, veterinary public health authorities) must make efforts to face and constrain this public health threat by applying safe clinic-to-home strategies. Veterinary clinics should continuously improve their infection prevention and control management and hygiene procedures to prevent animal patients and veterinary healthcare workers from developing infections or being colonized by CPE. They should establish surveillance based on hazard analysis and critical control points to determine the degree of environmental contamination and identify critical sources. Screening for CPE carriage in hospitalized companion animals must be established to inform owners and veterinary healthcare personnel [16]. Patients should inform their medical doctors that they own or work with animals since they may be at risk of carrying veterinary-acquired pathogenic bacteria, including CPE [17, 18, 31, 32]. Veterinary authorities should implement audits and provide support for surveillance. Any detection of CPE in animals, humans, or the environment should be reported to the responsible veterinary and public health authorities, and strains should be sent to national reference centers for rapid WGS-based comparative analysis. All sequences should be made available to open databases to facilitate outbreak investigation and traceability of the strains [33]. Related epidemiology and metadata should be confidentially recorded to rapidly inform people at risk in case of high clinic contamination burden and outbreaks.

In conclusion, the study by DeStefano et al underscores an alarming One Health concern from veterinary clinics that should be addressed immediately. In this setting, multidrug-resistant bacteria may evolve rapidly in different hosts and environments, making them better adapted for new niches and more capable of causing zoonotic infections. Incremental efforts across this and other sectors may contribute to curbing the rapid dissemination of CPE into human and animal populations and preventing the uncontrolled rise of avoidable infections.
